# Glucopyranosyl Lipid Adjuvant (GLA), a Synthetic TLR4 Agonist, Promotes Potent Systemic and Mucosal Responses to Intranasal Immunization with HIVgp140

**DOI:** 10.1371/journal.pone.0041144

**Published:** 2012-07-19

**Authors:** Mauricio A. Arias, Griet A. Van Roey, John S. Tregoning, Magdalini Moutaftsi, Rhea N. Coler, Hillarie P. Windish, Steven G. Reed, Darrick Carter, Robin J. Shattock

**Affiliations:** 1 Centre for Infection and Immunity, St. George’s University of London, London, United Kingdom; 2 Mucosal Infection and Immunity, Section of Infectious Diseases, Imperial College London, London, United Kingdom; 3 Infectious Diseases Research Institute (IDRI), Seattle, Washington, United States of America; Commissariat a l'Energie Atomique(cea), France

## Abstract

Successful vaccine development against HIV will likely require the induction of strong, long-lasting humoral and cellular immune responses in both the systemic and mucosal compartments. Based on the known immunological linkage between the upper-respiratory and urogenital tracts, we explored the potential of nasal adjuvants to boost immunization for the induction of vaginal and systemic immune responses to gp140. Mice were immunized intranasally with HIV gp140 together with micellar and emulsion formulations of a synthetic TLR4 agonist, Glucopyranosyl Lipid Adjuvant (GLA) and responses were compared to R848, a TLR7/8 agonist, or chitosan, a non TLR adjuvant. GLA and chitosan but not R848 greatly enhanced serum immunoglobulin levels when compared to antigen alone. Both GLA and chitosan induced high IgG and IgA titers in nasal and vaginal lavage and feces. The high IgA and IgG titers in vaginal lavage were associated with high numbers of gp140-specific antibody secreting cells in the genital tract. Whilst both GLA and chitosan induced T cell responses to immunization, GLA induced a stronger Th17 response and chitosan induced a more Th2 skewed response. Our results show that GLA is a highly potent intranasal adjuvant greatly enhancing humoral and cellular immune responses, both systemically and mucosally.

## Introduction

There is a general consensus that to prevent HIV infection an efficacious vaccine will need to induce both humoral and cellular immune responses. The generation of broadly neutralizing antibodies has been a goal of HIV vaccine research, but to date, efforts have largely been unsuccessful in both animals and humans inducing, at best, short-lived humoral responses that provide modest neutralization, most often limited to homologous virus and easy-to-neutralize (Tier 1) isolates [1]–[3]. The lack of broadly neutralizing antibodies following HIV vaccine trials is unsurprising, a number of broadly neutralizing antibodies have been isolated from humans [4], but these only develop in a very low proportion of them (>1%) after a period of many months to years [5]. Translating these findings into the development of immunogens capable of inducing similar responses in seronegative individuals poses a significant challenge where the timescale of discovery is uncertain [6], [7]. Nevertheless other effector functions such as viral aggregation (particularly by dimeric IgA), mucus trapping, inhibition of transcytosis, antibody dependent cellular cytotoxicity (ADCC), and Fc-mediated inhibition of infection may all enhance protection by antibody [8]. It is of note that V1/V2 binding antibodies and not neutralization were a correlate of protection in the RV144 trial [9] showing a modest protective efficacy, suggesting non-neutralizing antibodies may have had some impact on HIV acquisition.

Responses against HIV are predicted to be most effective at mucosal portals of HIV entry, since mucosal cellular responses may be required to control or eliminate the initial foci of infected cells. Previous human vaccine efficacy trials have focused on parenterally administered candidates which can induce response systemically and at mucosal sites. Systemic vaccination can induce mucosal immune responses, for example the HPV vaccine [10]. But mucosal immunization may alter the type of the mucosal immune response, in particular boosting IgA responses [11]. Our initial studies tested the efficacy of the vagina, the main site of infection, as an immunization site, but we observed that it was a poor site for the priming of antibody responses [12], [13]. Little is known about the induction of male genital tract immunity by parenteral or mucosal vaccines. Intranasal immunization may be a preferable option, due to the apparent immunological linkage between the upper-respiratory and urogenital tracts [14], the requirement for lower amounts of antigen than direct vaginal immunization and independence of the stage of the reproductive cycle [15] or patient gender. However, the impact of intranasal immunization on colorectal responses has been less well studies.

Despite the apparent advantages of intranasal immunization, there are no safe and effective mucosal adjuvants approved for human use. Indeed, widely accepted adjuvants such as Alum salts and MF59 used for systemic immunization are unsuitable for intranasal use due to their intrinsic generalized pro-inflammatory properties [16]. Bacterial toxins such as cholera toxin (CT) and heat-labile enterotoxin of *Escherichia coli* (LT) have been extensively studied as nasal adjuvants in mice [17] and humans [18], but may be of limited use in humans due to their potential neuronal binding and association with the induction of Bell’s palsy or facial nerve paralysis [19], [20]. Therefore, the identification of safe and effective intranasal adjuvants remains a critical gap for development of nasal vaccine strategies designed to induce urogenital antibody responses.

Formulated Toll-Like Receptor (TLR) agonists provide potent adjuvant candidates for mucosal use [21]. TLR4 agonists represent attractive candidates due to their good safety record associated with use in a number of parenteral adjuvants [22]. Triggering of TLR4 on the cell surface of antigen presenting cells is an important gateway for induction of innate and adaptive immune responses [23]. LPS, the prototype TLR4 ligand, is unsuitable for clinical use due to its high pyrogenicity, thus, modified products structurally related to LPS but devoid of high pyrogenicity and maintaining strong immunopotentiating characteristics have been developed. MPLA (monophosphoryl lipid A), a non-toxic derivative of LPS was the first TLR ligand and biological adjuvant approved for human use for its safety and effectiveness [24]. MPL is derived from acid hydrolysis of bacterial endotoxin, does not have the pyrogenic and toxic effects of LPS [25] and has been used for a number of human vaccines [26]. New generations of TLR4 agonists are being developed that are more receptor specific, possess less side effects but maintain or increase the adjuvant activity, including synthetic lipid A-like molecules such as glucopyranosyl lipid adjuvant (GLA) [27], [28] and aminoalkyl glucosaminide 4-phosphates (AGP) [29]. In contrast to MPLA, both AGP and GLA can be synthesized as highly pure, single chemical entities, and can be chemically modified in a defined manner to enhance their biological activity.

The aim of this work was to assess the potential of a micellar formulation of a synthetic TLR4 ligand, GLA-AF, to enhance systemic and mucosal immune responses to the gp140 HIV Ag after intranasal (i.n.) immunization. We compare the effects of a synthetic TLR4 ligand (GLA) to promote humoral immune responses to gp140 to that of a TLR7/8 synthetic ligand agonist Resiquimod (R848), and the non-TLR adjuvant chitosan. Our data indicate that GLA induced potent humoral and cellular immune responses to i.n. immunization with gp140 in local and distant mucosas, as well as in the systemic compartment. These data suggest that GLA-AF may represent an important new adjuvant for mucosally delivered HIV vaccines.

## Materials and Methods

### Animals

Female BALB/c or C57BL/6 mice, 6–8 week old, were obtained from Harlan Olac Ltd (Landue, UK), Charles River (Wilmington, MA) or Jackson Laboratories (Bar Harbor, ME). All procedures were performed in accordance with the United Kingdom’s Home Office standards under the Animals Scientific Procedures Act, 1986, and approved by the School’s Ethical Review Committee or IDRI Institutional Animal Care and Use Committee (IACUC).

### Antigen and Adjuvants

A clade C HIV-1 envelope clone p97CN54 was originally isolated from a Chinese patient [30] and was made available by H. Wolf and R. Wagner, University of Regensburg, Germany. Trimeric gp140 (gp120 plus the external domain (ED) of gp140), designated CN54 gp140, was produced as a recombinant product in CHO cells and manufactured to GMP specification by Polymun Scientific, Vienna, Austria.

The micellar formulation of GLA has been denoted previously as IDRI-AQ001 [31] and the emulsion as EM005 and are more generally denoted as GLA-AF and GLA-SE respectively. Biological and physicochemical characterization of GLA has been published previously [28]. R848 was used as an aqueous formulation and has been previously denoted as IDRI-RM002. Both adjuvant systems were obtained from the Infectious Diseases Research Institute (IDRI, Seattle, U.S.). Protasan UP G 213 (FMC BioPolymer, AS, Novamatrix, Norway) is a chitosan glutamate where 75–90 percent of the acetyl groups are deacetylated. The cationic polymer is a high molecular weight (200,000–600,000 g/mol) chitosan with very low levels of endotoxin and manufactured under GMP, it contains ≤100 EU/g endotoxin.

### Immunization Protocol

Mice were immunized 2–3 times with 3 weeks interval, with 10–20 µg gp140, with or without adjuvants: 10 µg GLA-AF and R848, and 100 µg chitosan in a maximum volume of 25 µl. The formulation was gently dispensed in the animal’s nostrils after isofluorane (BALB/c) or ketamine-induced anesthesia (C57BL/6). Intramuscular immunizations were delivered in two 50 µl volumes, one per leg.

### Sample and Tissue Collection

Serum and mucosal samples were obtained at pre-immunization and 21 days after each immunization as described previously [32]. Nasal washes were obtained at the end of the experiment.

Splenocytes were isolated by mechanical dissociation through sterile nylon mesh, followed by red blood cell lysis. Cells were always kept on ice unless specified otherwise.

The mouse genital tract, including vagina, uterus, oviducts, and ovaries were dissected from the animal, and placed in cold CM (RPMI-1640 supplemented with 10% fetal bovine serum (FBS), 2 mM glutamine, 10 mM HEPES, 100 IU/ml penicillin, 100 µg/ml streptomycin, and 10 µg/ml gentamycin). The tissue was finely cut with a scalpel, washed with CM and digested at 37°C for 1 h on a shaker with 5 ml of serum-free RPMI-1640 medium that contained 2 mg Collagenase Dispase and 0.1 mg/ml DNaseI (Roche Diagnostics). The digested tissue was spun and the cell pellet washed twice in CM. The lymphoid cell population was separated from the stromal cells by density gradient centrifugation (Lympholyte, Cedarlane Laboratories).

### ELISA

HIV gp140 specific antibody ELISA were performed as described previously [32]. In brief, plates were coated overnight with 5 µg/ml gp140 in PBS. Serially diluted samples were incubated for 1 hr at 37°C prior to detection with Ig isotype specific secondary antibody. To increase IgA sensitivity biotin-Streptavidin HRP amplification step was used. Plates were developed with TMB (Pierce) prior to quenching with H_2_SO_4_ and reading at 450 nm. A mix of pre-immune samples was run in 6 replicates per plate, and the cut-off for titer calculation calculated (after subtracting the blank) as the mean of these 6 values plus 3 SD, except for that of feces where 5 SD were used.

### ELISPOT

Cells from genital tract, bone marrow and spleen were assessed for the presence of gp140-specific IgG and IgA antibody secreting cells (ASC). Cells from genital tract were assessed immediately after isolation, whereas the splenocytes were cultured for 72 h at 10^6^ cells/ml in the presence of 20 µg/ml IL-2 (R&D Systems) and 10 µg/ml pokeweed mitogen (Sigma-Aldrich). ELISPOT assays were performed using a commercial kit from MABTECH (Nacka Strand, Sweden) following the manufacturer’s recommendations. The spots were counted using the AID ELISPOT reader ELR03 (Autoimmune Diagnostika).

### Cell Proliferation Assay

#### [^3^H] Thymidine incorporation

Splenocytes (2×10^5^ cells) were cultured for 5 days with or without 5 µg/ml gp140 in CM and 5 µg/ml Con-A from Canavalia ensiformis used as a positive control. Supernatants were collected at 48 h for assessment of cytokine release by Luminex (R&D). Cells were pulsed with 0.5 µCi [^3^H]Td/well 18 h before harvesting, and c.p.m. determined in a liquid scintillation β counter (1450 Microbeta Plus, Wallac Oy). Proliferation response was expressed as stimulation index (SI), calculated by dividing the c.p.m. of the experimental by the c.p.m. of the non-stimulated cells (medium alone).

### Detection of Cytokines by Multiplex Assay (Luminex)

Mouse IL-4, IL-17, and IFN-γ in cell culture supernatants were detected using a multiplex assay Luminex kit, following the manufacturer’s protocol (R&D).

### Flow Cytometry

All flow cytometry reagents were from BD unless otherwise stated. For both CFSE and intracellular staining, fifty thousand events per sample were acquired on a BD FACSCanto II and analyzed using the BD FACSDiva software version 6.1.3. Background fluorescence and color compensation was performed using Anti-Rat Ig beads (BD CompBeads) as described by the manufacturer. Analysis was performed on the CD3+ T-cell population by gating on live cells detected by Forward and Side scatter. Subgating for CD4+ and CD8+ cells from the CD3+ gate was subsequently performed and expressed as a function of CFSE staining on the green channel, for proliferation analysis, or as a function of cytokine-specific fluorochrome-antibody labeled for cytokine analysis.

### CFSE Assay

Splenocytes were stained with 2 µM CFSE for 10 min at 37°C. The cells were then cultured for 5 days with or without 5 µg/ml gp140 in CM. Con-A was used at 5 µg/ml as a positive control of stimulation. Cells were immunostained with 1 µg/ml per million cells of labeled rat anti-mouse PE-Cy7-CD3, allophycocyanin (APC) H7-CD8, and APC-CD4. The cells were then stained with violet fluorescent reactive dye (Live/dead fixable dead cell stain kit, Invitrogen, Paisley, UK) to separate live from dead cells by flow cytometry. The proportion of cells per proliferation cycle in relation to the whole number of gated CD4+ or CD8+ was calculated and plotted comparatively.

### Intracellular Cytokine Staining

Cells were stimulated for 6 hours with a CN54 peptide pool plus 2 µg/ml CD28 in the presence of GolgiStop. Cells cultured in medium alone or with 50 ng/ml PMA plus 500 ng ionomycin were used as negative and positive control of stimulation, respectively. Peptides were selected from a bigger pool of 156 peptides covering the whole of HIV-1 CN54 gp140 for their ability to restimulate splenocytes in vitro after immunization. Cells were labeled for mouse surface antigens using Pacific Blue anti CD3e (BD), V500 anti- CD4 (BD), PerCP anti- CD8a (BioLegend), and blocked using purified rat anti-mouse CD16/CD32 (BD). After staining, cells were washed, fixed and permeabilised using BD Fixation/Permeabilisation Kit. Intracellular cytokine staining was accomplished using PE-Cy7 anti-mouse IL-4, PE anti-mouse IL-17A, allophycocyanin-Cy7 anti-mouse IL-2, FITC anti-mouse TNF-α and allophycocyanin anti-mouse IFNγ (All BD). Appropriate isotype controls were used in all steps.

### Statistical Analyses

Analyses were performed using GraphPad Prism, version 4.00 (GraphPad Software). Data was expressed as the mean ± SEM. Statistical differences between 3 or more groups were calculated by one-way ANOVA with Tukey’s multiple comparison posttest to compare groups by pairs, or Dunn’s multiple comparison test when analyzing non-parametric data. Differences between groups in relation to time were analyzed by two-way ANOVA, with Bonferroni’s posttest for comparison of pairs. Unpaired Student’s *t* test was used to compare the means of two groups. Differences were considered significant at p≤0.05.

## Results

### Different Formulations of GLA Enhance Humoral Immune Responses to gp140

**Figure 1:**
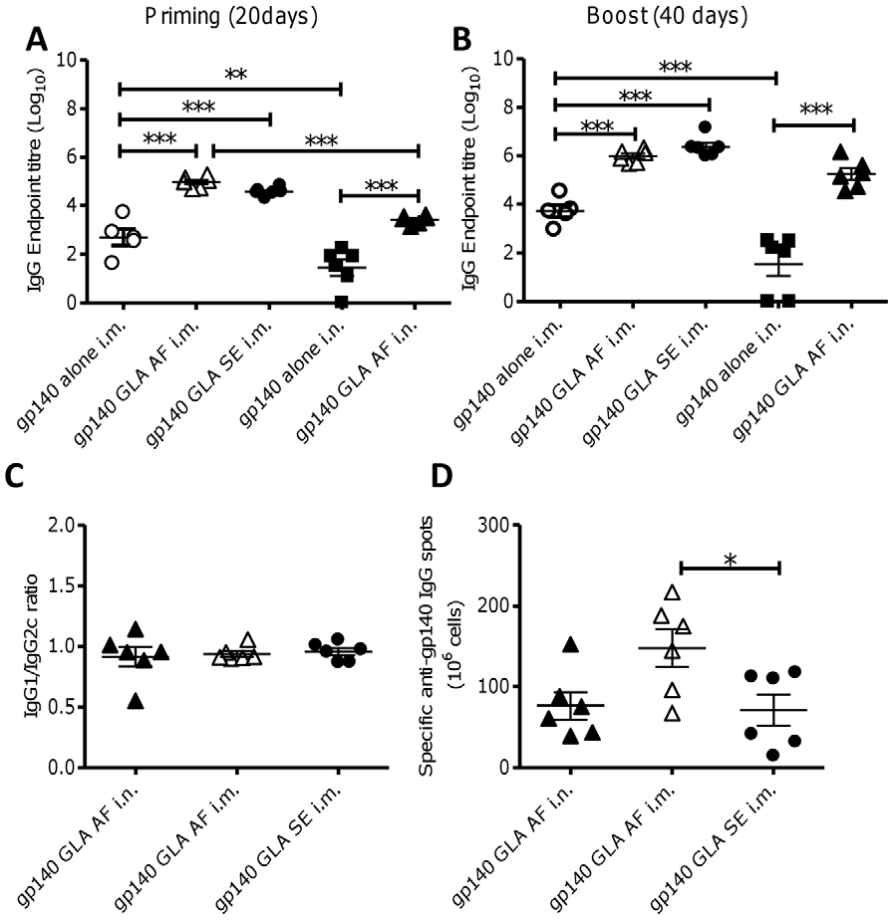
Micellar GLA is a potent adjuvant for HIV gp140. C57Bl/6 mice were immunized with 10 µg HIV gp140 antigen alone or in the presence of different formulations of GLA by the intramuscular (i.m.) or intranasal (i.n.) routes. Micellar GLA (GLA-AF) was given i.m. (Δ) or i.n. (▴) and emulsion GLA (GLA-SE: •) was given i.m., responses were compared to gp140 alone i.n. (▪). Anti-gp140 IgG was measured in sera of mice after prime (A) or boost (B) immunization. HIV gp140 specific IgG subtypes (C) and bone marrow long-lived plasma cells (D) were measured after 2 immunizations. Bars represent mean of n = 6 mice ± SEM, *p<0.05, **p<0.01, ***p<0.001.

Initial experiments were performed to determine whether a micellar formulation of GLA (GLA-AF) could enhance serum IgG responses to gp140 that were comparable to those promoted by the emulsified form (GLA-SE) via the intramuscular route. In addition, intranasal (i.n.) immunization was compared to intramuscular (i.m.) immunization, because the induction of mucosal immune responses to gp140 are highly relevant to the development of a protective HIV vaccine. GLA-SE was not assessed for i.n. immunization as emulsions are likely to be less compatible for i.n. use. Animals were immunized two times at 3 week intervals, and sampled before (d20) and after (d40) boosting immunization to test for specific IgG production. The addition of GLA significantly increased antibody responses compared to antigen alone, regardless of route or formulation (p<0.001, Fig 1A, B). After priming, intranasal immunization with GLA-AF i.n. induced significantly higher IgG titers than antigen alone (p<0.001, Fig 1A), but lower than the i.m. route. However after boosting the titer was indistinguishable to the i.m. route (Fig 1B). By the i.m. route GLA-AF was indistinguishable from GLA-SE after priming (Fig 1A) or boosting (Fig 1B). A balanced Th1/Th2 response was observed regardless of the route of immunization, based on levels of IgG1 and IgG2c (Fig 1C). The adjuvant effect of GLA-AF was derived from the GLA and not the excipients in the micellar formulation, as immunization with gp140 plus formulation alone had no effect on antibody titer (data not depicted). Both i.m and i.n immunization with gp140 plus GLA-AF induced homing to the bone marrow of gp140-IgG-specific long-lived memory B-cells (Fig 1D). From these studies we conclude that the micellar formulation of GLA (GLA-AF) is as effective an adjuvant as the emulsion (GLA-SE) in this model and can be used intranasally to induce an immune response.

### The use of GLA as an Adjuvant Induces Stronger Humoral Responses than Chitosan or R848

**Figure 2:**
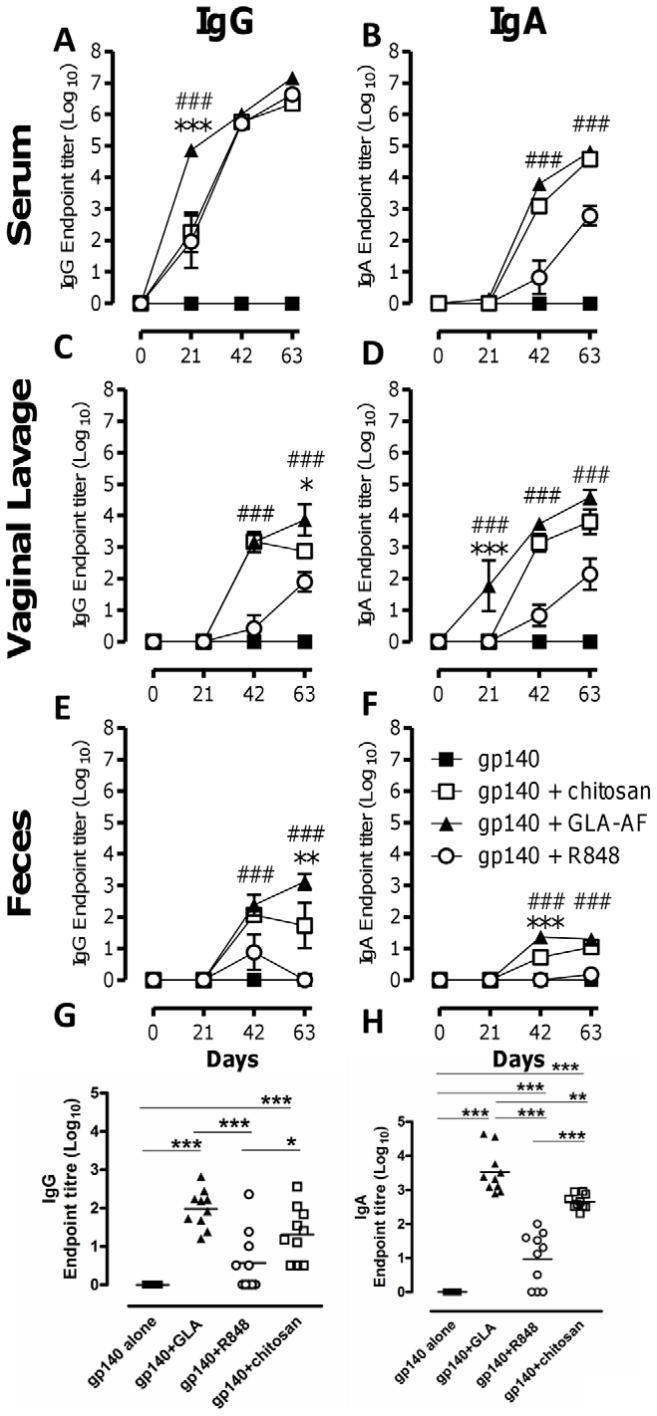
GLA is a more potent mucosal adjuvant than R848 or chitosan. BALB/c mice were immunized i.n. three times at 3 week intervals with 10 µg gp140 and 10 µg GLA (▴), 10 µg R848 (○) or 100 µg chitosan (□) compared to antigen alone (▪). Serum (A, B), vaginal lavage (C, D), feces (E,F) were collected after each immunization and nasal lavage collected at d 63 only (G, H). gp140 specific IgG (A, C, E, G) and IgA (B, D, F, H) were measured by ELISA. Points represent mean of n = 10 mice ± SEM, *p<0.05, **p<0.01, ***p<0.001 comparing GLA and chitosan; #p<0.05, ##p<0.01, ###p<0.001 comparing GLA and R848.

A core aim of our studies was to induce mucosal responses to HIV and since we observed that i.n. immunization could induce serum antibody, we wished to test the effect of administration of GLA-AF by this route on mucosal responses. The adjuvant effect of GLA was compared with another TLR ligand, R848, which binds TLR7 in mice (and TLR7/8 in humans), and the polycationic polysaccharide chitosan, a recognized muco-adherent product used in the intranasal delivery of vaccines [33]. To allow comparison with our previous studies using gp140, particularly T cell protocols [34], these studies were performed in BALB/c mice. Serum IgG levels in BALB/c mice following i.n. prime/boost immunization were similar on day 42 (Fig 2A) compared to C57Bl/6. Animals were immunized three times at 3 week intervals, and sampled before priming and 3 weeks after every immunization to test for the magnitude and kinetics of specific IgG and IgA production in serum, vaginal lavage and feces.

Intranasal immunization with gp140 plus GLA, R848 or chitosan induced significantly higher titers of IgG in the serum than antigen alone from d21 (p<0.001, Fig 2A). GLA induced IgG more rapidly and to a significantly greater level than R848 and chitosan (p<0.001) on d21, but by d42 IgG levels in sera were similar in all three groups. It is of note that significant IgG responses were observed after a single dose of GLA-gp140 (d21). All three adjuvants induced higher sera IgA levels than antigen alone by d63. There was no difference between GLA and chitosan and the responses to both these adjuvants began earlier and peaked higher than R848. GLA induced significantly higher levels of IgA than R848 (p<0.001, Fig 2B) following both the first (d42) and second boost (d63).

All three adjuvants led to detectable antibody responses at mucosal surfaces. GLA induced significantly higher levels of IgG (p<0.05, Fig 2C) in the vaginal lavage than either chitosan or R848 on day 63; and chitosan induced a higher level of mucosal IgG than R848 at d42 (p<0.001) and d63 (p<0.05). GLA induced significantly greater levels of vaginal lavage IgA than R848 at all time points (p<0.01, Fig 2D) and chitosan on d21 (p<0.05) but not subsequent time points and chitosan induced higher responses than R848 at d42 and d63 (p<0.01). A similar, but reduced pattern was seen in the feces (Fig 2E and 2F). While specific IgG titers in feces following GLA-gp140 immunization steadily increased over time, titers induced by chitosan-gp140 or R848-gp140 decreased after the second immunization (Fig 2E). Specific IgA was detected in feces after GLA-gp140 and chitosan-gp140 but not R848-gp140 immunization (Fig 2F). Specific IgG and IgA responses were also assessed in nasal lavage at the end of the experiment; GLA, R848, and chitosan all promoted gp140-specific IgG and IgA responses (Fig 2G and H). In line with the other mucosal sites, GLA promoted higher IgG and IgA responses to gp140 than chitosan, which in turn promoted higher responses than R848. These results suggest that GLA strongly promotes gp140-specific IgG and IgA responses both in the systemic and mucosal compartments following intranasal immunization and that it is induced more rapidly (single-immunization) and sustained longer than chitosan or R848.

### GLA Promotes gp140-specific IgG and IgA ASC in Spleen and Genital Tract after Intranasal Immunization

**Figure 3:**
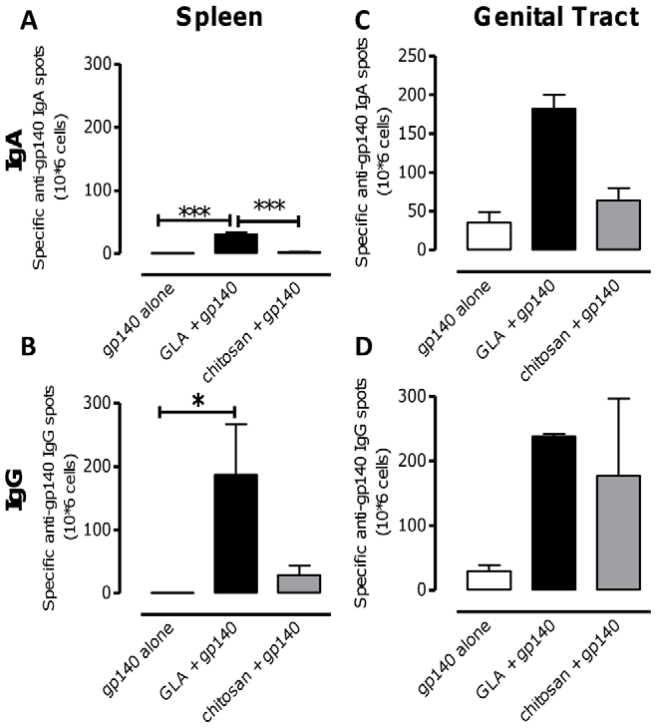
GLA boosts gp140 specific B cell numbers in spleen and female genital tract. Mice were immunized i.n. three times at 3 week intervals with 10 µg gp140 alone, with 10 µg GLA or 100 µg chitosan. Three weeks after the third immunization spleens were removed and gp140 specific IgA (A) and IgG (B) antibody secreting cell (ASC) numbers assessed by ELISPOT on splenocytes stimulated for 3 days with IL-2 plus pokeweed mitogen. Bars represent mean of n = 5 mice ± SEM, *p<0.05, **p<0.01, ***p<0.001. gp140 specific IgA (C) and IgG (D) antibody secreting cell (ASC) numbers assessed by ELISPOT on cells from female genital tract immediately after isolation. Bars represent mean of 2 pools of female genital tract cells, cells were pooled from either 2 or 3 mice.

Since GLA and chitosan promoted stronger anti-gp140 responses than R848 we decided to further investigate the nature of the responses to these adjuvant regimes. B-cell ELISPOT assays were performed to determine specific IgG and IgA antibody-secreting cells (ASC) in the spleen and genital tract after i.n. immunization with gp140. Splenocytes were stimulated for 3 days with IL-2 plus pokeweed mitogen to increase the likelihood of detecting specific ASC. To normalize the numbers of specific IgG and IgA ASC for each treatment, total IgG and IgA ASC were assessed in parallel. Intranasal gp140 plus GLA induced significantly higher numbers of gp140-specific IgA (p<0.001, Fig 3A) and IgG (p<0.05, Fig 3B) ASC in the spleen than gp140 alone. These differences were clearly detected before and after normalization against total IgA ASC (data not depicted). Female genital tracts were collected and pooled (due to low cellular yields) from mice, following isolation by collagenase digestion and density gradient purification of lymphocytes. Cells from genital tract were assessed by ELISPOT for specific gp140 IgG and IgA secretion immediately after isolation. Intranasal GLA-gp140 induced higher numbers of gp140-specific IgA ASC in the genital tract than antigen alone or antigen plus chitosan (Fig 3C), but numbers of gp140-specific IgG ASC were similar between groups (Fig 3D). These results show that GLA can promote the induction of high numbers of gp140-specific IgG and IgA ASC both in spleen and genital tract after intranasal immunization with gp140.

### T Cell Responses Following the use of GLA or Chitosan as Adjuvants

**Figure 4:**
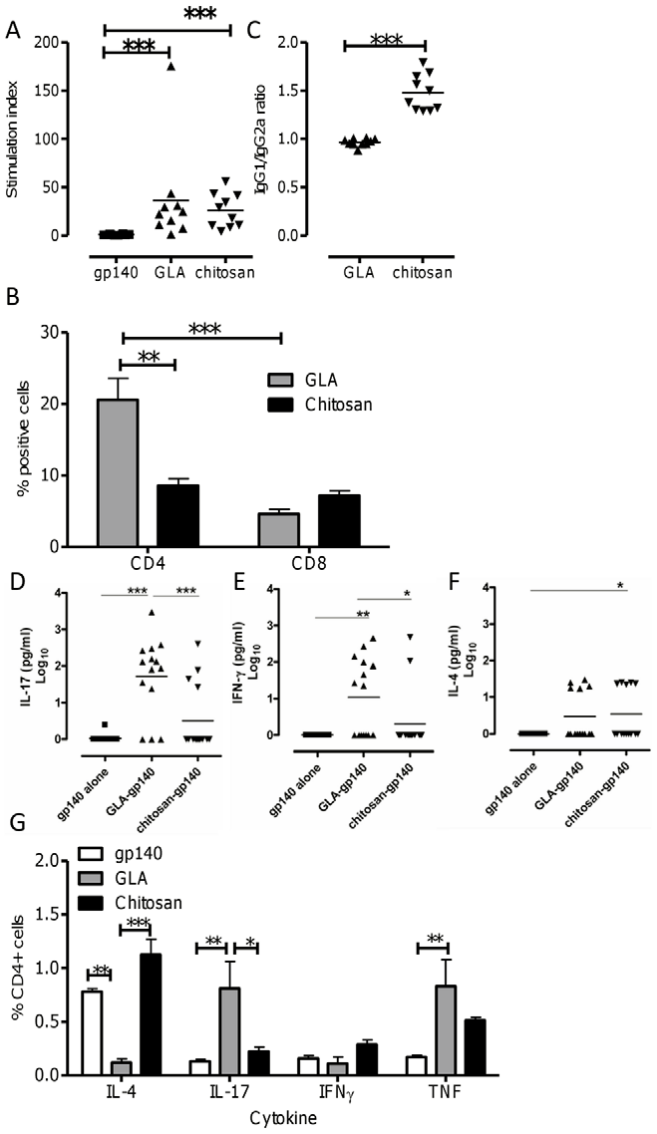
GLA boosts T cell responses and switches to a Th17 response. Mice were immunized 3 times with 10 µg gp140 alone or with 10 µg GLA or 100 µg chitosan. T cell proliferation responses in spleens were measured by [^3^H] Thymidine incorporation (A) or CFSE dilution (B) after the third immunization. Antigen specific IgG subtype responses were measured in sera (C). IL-17 (D), IFNγ (E) and IL-4 (F) were analyzed in supernatants from T cell proliferation assays by Luminex. Antigen specific T cell cytokine responses were measured by intracellular staining of gp140-peptide stimulated T cells (G). Bars represent mean of n = 5 mice ± SEM, *p<0.05, **p<0.01, ***p<0.001.

The ability of GLA and chitosan to enhance T-cell responses to gp140 following i.n. immunization was assessed. Intranasal immunization with gp140 and GLA or chitosan induced very strong antigen specific proliferation responses when compared to gp140 alone. No differences were observed between the effect of GLA and chitosan on gp140 specific T-cell proliferative responsiveness (Fig 4A). To determine the predominant proliferating T-cell type, a CFSE dilution assay was used. The use of GLA led to a greater gp140 specific CD4+ T-cell response (p<0.001), in contrast to a balanced CD4+ and CD8+ T-cell proliferation response to gp140 when chitosan was used as an adjuvant (Fig 4B). Comparing the two adjuvants, gp140 specific CD4 responses were significantly greater after GLA-gp140 than chitosan-gp140 (p<0.01), but CD8 responses were broadly similar. These results show that i.n. gp140 delivered with both GLA and chitosan enhance T-cell proliferation, with GLA leading to a stronger CD4 T cell response.

We wished to determine whether the difference in CD4 proliferation was caused by a difference in the quality of T helper responses. Whilst GLA gave a balanced response, the use of chitosan significantly biased the antibody response towards IgG1, indicating a Th2 bias (p<0.001, Fig 4C). To investigate the differences in cellular responses, the cytokines IL-4, IFNγ, and IL-17 were assessed by a multiplex assay on the cell culture supernatants obtained from the T-cell proliferation experiments. Levels of both IL-17 (p<0.001, Fig 4D) and IFNγ (p<0.05, Fig 4E) were significantly higher following the use of GLA as an adjuvant. The use of chitosan as an adjuvant led to significantly higher levels of IL-4 than antigen alone (p<0.05, Fig 4F). In parallel, splenocytes were stimulated with a gp140 peptide pool and the intracellular expression of the cytokines IFNγ, IL-4, TNF and IL-17 by CD4+ T cells assessed by flow cytometry (Fig 4G). Following immunization with GLA, significantly more IL-17+ (p<0.01) and TNF+ (p<0.01) CD4^+^ T cells were detected and immunization with chitosan significantly increased the proportion of IL-4 secreting CD4 T cells (p<0.01). Of note, Th17 cells were only induced following i.n. but not i.m. immunization with GLA (data not depicted). Levels of IFNγ-producing CD4T cells were similar following both regimes and significantly greater than antigen alone.

## Discussion

Here we show that a formulated, synthetic TLR4-based adjuvant, GLA-AF can be used to boost vaccine responses to nasally delivered antigens. GLA appears to be more potent than chitosan or R848, inducing antibody responses that appear earlier and are of a greater magnitude. Both GLA and chitosan were able to induce specific antibody and B cell responses at distant mucosal sites following intranasal immunization. In addition to boosting antibody responses, both GLA and chitosan boosted T cell responses, with GLA skewing responses to a Th17 type response and chitosan skewing responses to Th2.

This is the first study to show that GLA is an effective adjuvant when delivered mucosally. When used as a parenteral adjuvant, formulations of GLA have been shown to generate strong immune responses to influenza [31], [35], malaria [23], HIV [36] and TB [37], [38] antigens in mice, with no obvious detrimental side effects. In the current study GLA induces a slightly more potent antibody response than chitosan and a greatly more potent response than the R848 formulation used in this study. In part, this may be a feature of murine TLR biology and although mice respond to R848 through TLR7, they do not express functional TLR8.

GLA administration upregulates the expression of CD86 and CD40 on DC and induces IL-12p70 [36], with a similar immunostimulatory profile to formulations of the naturally derived TLR4 ligand MPLA, although with greater potency [27]. The commercial adjuvant, AS04, contains alum and MPLA and it has been demonstrated that the adjuvant effect of AS04 is principally due to the MPLA component [39]. It has also been observed that the addition of GLA to an oil in water adjuvant was critical for protective responses [40]. The formulation of the adjuvant is important, the addition of alum to the AS04 adjuvant may serve to prolong responses and previous studies have shown that T cell responses were higher when GLA was formulated in an emulsion compared to the micellar form and delivered intradermally [41], however here we show that for HIV gp140, the micellar formulation gives equivalent responses and can be delivered intranasally.

In a similar fashion to GLA, high levels of HIV-1 gp140 specific IgA and IgG were detected in serum, vaginal lavage, and feces, when chitosan was used as an adjuvant. Chitosan has been shown to be an effective adjuvant when delivered nasally with influenza antigens [42], but the mechanism is not known. The adjuvant effect of chitosan seen here may be due to the opening of epithelial tight junctions, increasing permeability of the nasal mucosa thus enhancing diffusion of HIV gp140 into the submucosal compartment [43]. Alternatively, the viscosity of the chitosan may help to keep the antigen at the epithelial surface for a longer time, avoiding inhalation into the lungs or swallowing. It has also been suggested that chitosan can enhance immune responses by activating natural killer cells, macrophages, and DC [44]. To test its effect on pattern recognition receptors, we have screened chitosan against a panel of TLR and NLR cells and have found that it has no reactivity (data not depicted) suggesting that it works by increasing epithelial permeability or by acting as an antigen depot due to its particulate nature.

In contrast to the antibody responses, we saw differences in the T cell response following the administration of different adjuvants. The use of GLA induced a greater CD4 T cell proliferation than chitosan and the response after intranasal GLA was pro-inflammatory (TNF) and Th17 skewed, whilst chitosan induced a more Th2 phenotype. When applied to human monocyte-derived DC, GLA has been shown to induce the upregulation of IL-6 [27], which may explain the Th17 switch observed in the current study. Likewise, house dust mite allergen induces a TLR4 dependent pro-Th17 phenotype in lung DCs, upregulating IL-6 and IL-23 [45]. It is not clear why this switch to Th17 only occurred following i.n. but not i.m. immunization - previous studies have shown that intramuscular delivery of GLA drives a Th1 skewing- but the context of antigen exposure often shapes cellular responses. As described above, chitosan’s mechanism of action is not known but it is of note that the T cell response is more Th2 skewed. The Th2 skewing may be associated with its particulate nature: other microparticles such as alum are also pro-Th2 [16] and other studies have demonstrated that changing particle size [46] or route of immunization [34] can change the T helper bias of responses. It remains to be seen what effect T helper skewing has on the protective efficacy of an HIV vaccine and it is of note that the GLA induced TNF and IL-17 producing CD4 T cells which may promote HIV replication. We have previously described the use of the pro-Th2 cytokine, TSLP, as a nasal adjuvant [32] and hypothesized that the absence of pro-inflammatory T cells at mucosal sites may be advantageous as they can act as targets for HIV infection.

Here we show that the TLR4 agonist GLA is a potent adjuvant when delivered intranasally. It is striking that it has a pro-Th17 effect when delivered by this route. Emerging evidence suggests that Th-17 cells are critical regulators of host defense against bacterial, fungal, and viral infections at mucosal surfaces as well as having critical roles in inflammation and autoimmunity [47]. The role of Th17 responses in immunity to mucosal acquired HIV-1 infection is unclear, however progressive HIV disease is associated with preferential depletion of CD4 Th17 cells [48]–[50]. Taken together, our data show that GLA-AF is a highly potent intranasal adjuvant greatly enhancing humoral and cellular immune responses to HIV gp140 both systemically and mucosally. It will be important to determine the adjuvanticity of GLA for gp140 when delivered intranasally to NHP or humans.
